# N_2_O dynamics in the western Arctic Ocean during the summer of 2017

**DOI:** 10.1038/s41598-021-92009-1

**Published:** 2021-06-15

**Authors:** Jang-Mu Heo, Seong-Su Kim, Sung-Ho Kang, Eun Jin Yang, Ki-Tae Park, Jinyoung Jung, Kyoung-Ho Cho, Ju-Hyoung Kim, Alison M. Macdonald, Joo-Eun Yoon, Hyo-Ryeon Kim, Sang-Min Eom, Jae-Hyun Lim, Il-Nam Kim

**Affiliations:** 1 Department of Marine Science, Incheon National University, Incheon, 22012 South Korea; 2 Korea Polar Research Institute, Incheon, 21990 South Korea; 3 Faculty of Marine Applied Biosciences, Kunsan National University, Gunsan, 54150 South Korea; 4 Physical Oceanography Department, Woods Hole Oceanographic Institution, MS 21, 266 Woods Hold Rd., Woods Hole, MA 02543 USA; 5 Fisheries Resources and Environmental Research Division, East Sea Fisheries Research Institute, National Institute of Fisheries Science, Gangneung, 25435 South Korea

**Keywords:** Biogeochemistry, Ocean sciences

## Abstract

The western Arctic Ocean (WAO) has experienced increased heat transport into the region, sea-ice reduction, and changes to the WAO nitrous oxide (N_2_O) cycles from greenhouse gases. We investigated WAO N_2_O dynamics through an intensive and precise N_2_O survey during the open-water season of summer 2017. The effects of physical processes (i.e., solubility and advection) were dominant in both the surface (0–50 m) and deep layers (200–2200 m) of the northern Chukchi Sea with an under-saturation of N_2_O. By contrast, both the surface layer (0–50 m) of the southern Chukchi Sea and the intermediate (50–200 m) layer of the northern Chukchi Sea were significantly influenced by biogeochemically derived N_2_O production (i.e., through nitrification), with N_2_O over-saturation. During summer 2017, the southern region acted as a source of atmospheric N_2_O (mean: + 2.3 ± 2.7 μmol N_2_O m^−2^ day^−1^), whereas the northern region acted as a sink (mean − 1.3 ± 1.5 μmol N_2_O m^−2^ day^−1^). If Arctic environmental changes continue to accelerate and consequently drive the productivity of the Arctic Ocean, the WAO may become a N_2_O “hot spot”, and therefore, a key region requiring continued observations to both understand N_2_O dynamics and possibly predict their future changes.

## Introduction

Arctic air temperatures have dramatically increased over the past two decades. This strong warming is frequently referred to as “Arctic Amplification” and is one of the main results of rapidly increasing concentrations of greenhouse gases in the atmosphere^1−4^. Consequently, the extent of annual sea ice has rapidly decreased since the 1980s^5−7^, implying that we may face a sea-ice-free Arctic summer in the near future^8^. The western Arctic Ocean (WAO) has also experienced rapid environmental changes, such as increased heat transport and sea-ice reduction^9,10^.

**Figure 1:**
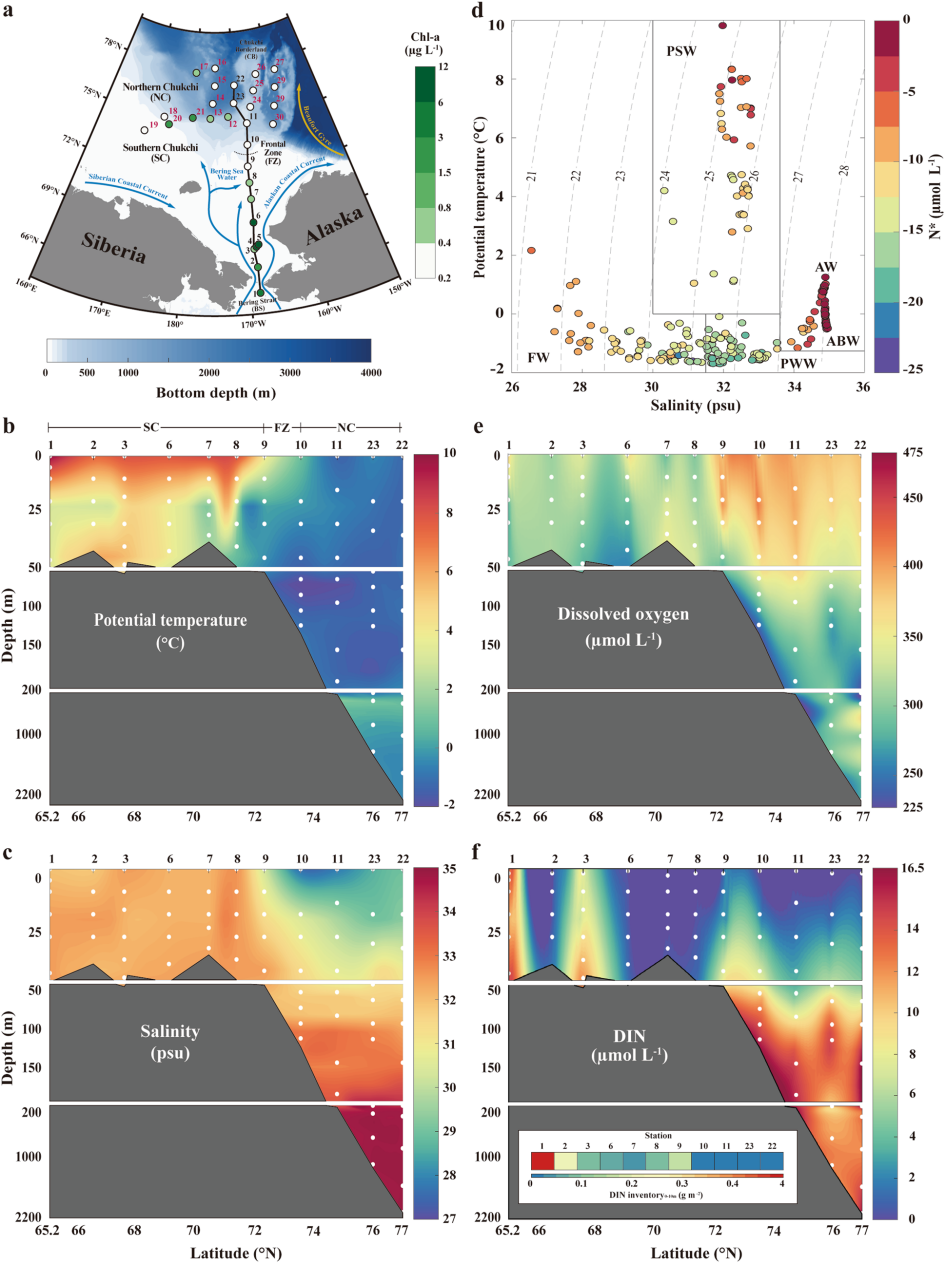
Study area map and physicochemical properties of WAO. (**a**) Map of sampling stations using the Ice Breaking R/V Araon during August 2017 with bathymetry information (a horizontal white‒blue gradient color bar). The sampling locations were filled with chlorophyll-*a* concentrations (white to green colors). In this study, Sts. 1‒9 are located in the SC (i.e., Bering Strait to Chukchi Shelf), and Sts. 10‒30 are placed in the NC (i.e., Chukchi Borderland and Canada Basin). The FZ is between St. 9 and 10 (black dotted line). Schematic arrows represent major surface currents (blue) and gyres (yellow) identified in the study area during the summer: Siberian Coastal Current, Alaskan Coastal Current, Bering Sea Water, and Beaufort Gyre. Vertical distributions of (**b**) potential temperature (°C), and (**c**) salinity (psu) along a latitudinal transect from the Bering Strait to the Chukchi Borderland (black solid line shown in **a**). (**d**) Potential temperature‒salinity diagram with N^*^ information (blue to red gradient color bar); vertical distributions of (**e**) dissolved oxygen (μmol L^−1^) and (**f**) dissolved inorganic nitrogen (DIN; μmol L^−1^). The inset in (**f**) shows the DIN inventory (g N m^−2^) between the surface and 10 m at each station (red to blue gradient color bar). Note that this figure was generated using MATLAB program (ver. R2019b and www.mathworks.com).

The WAO is geographically composed of the Chukchi, East Siberian, and Beaufort Seas, the Canadian Arctic Archipelago, and the Canada Basin (Fig. 1a). During the summer season, latitudinal differences in both physical and biogeochemical features have been clearly determined from the Bering Strait to the Chukchi Borderland^11−13^. The southern region (i.e., the area extending from the Bering Strait to the Chukchi Shelf) is relatively warm, saline, and eutrophic due to the presence of Pacific waters that enter the WAO, bringing heat and nutrients with them. As a result, this region is one of the most productive stretches of ocean in the world^14,15^. By contrast, the northern region, extending from the Chukchi Borderland to the Canada Basin, is mainly affected by freshwater originating from sea ice melt and rivers, and is therefore relatively cold, fresh, and oligotrophic. It is also important to note that latitudinal variations in the distribution of bacterial communities within the WAO can be caused by both physical and biogeochemical factors^12^. These environmental variations are therefore extremely relevant to nitrous oxide (N_2_O) dynamics.

N_2_O production through marine nitrogen cycle processes (i.e., nitrification and denitrification) is directly linked to climate change, contributing to both the greenhouse effect and ozone depletion^16,17^. Although it has also been reported that a substantial portion of the N_2_O budget may be effluxed from the global ocean (4.4 ± 0.7 Tg N year^**−**1^), N_2_O data are still limited, particularly over much of the Arctic Ocean^18^. That said, several N_2_O investigations in the WAO have previously been conducted and have provided important information regarding N_2_O dynamics^13,19−25^. In particular, high N_2_O concentrations were observed in the Chukchi Sea shelf region, whereas most of the under-saturated N_2_O was found in the high-latitude regions of the WAO. However, prior to the study presented here, no intensive investigation synthesizing information on the dynamics of WAO N_2_O (i.e., distributions of the concentration and flux and their controlling environmental factors) in the water column (from the surface to the bottom) had been conducted.

The concentration of N_2_O in the atmosphere has been steadily increasing since pre-industrial times, and because the points in time at which the N_2_O concentration of water parcels were in equilibrium with the atmosphere prior to being ventilated are all different, it is necessary to calculate equilibrium N_2_O concentration to accurately estimate the amount of N_2_O production. Although there have been a few studies attempting to accurately calculate the N_2_O concentration of the water column^20,22,25^, the N_2_O concentration of the water column in other studies has been calculated from the contemporary atmospheric N_2_O concentration^13,19,21^. Hence, in this study, the equilibrium N_2_O concentration was calculated using a tracer gas.

Based on an intensive and precise N_2_O survey of the WAO water column during the open-water season of summer 2017, we (1) present spatial distributions of N_2_O concentrations and fluxes, (2) identify physical and/or biogeochemical factors controlling the distributions, (3) determine whether the WAO is a source or sink for atmospheric N_2_O, and (4) speculate regarding future changes in the WAO N_2_O flux in response to rapid Arctic climate changes.

## Materials and methods

### Sampling and measurement of basic physical/biogeochemical parameters

In August 2017, the ice breaker R/V Araon collected physical and biogeochemical samples from 30 WAO stations from the Bering Strait to the Chukchi Borderland (Fig. 1a). At each sampling location, the vertical profiles of the potential temperature (θ), salinity (S), and dissolved oxygen (DO) were measured using a conductivity-temperature-depth instrument (CTD; SBE 911 plus, Sea-Bird Electronics, Inc., USA). The CTD temperature and conductivity accuracies were ± 0.001 °C and ± 0.0003 S m^**−**1^, respectively (sensor specifications can be found at https://seabird.com/). The CTD salinity measurements were calibrated with discrete bottle samples analyzed using a laboratory salinometer (Model 8400B, Guideline Instruments, Canada)^26^. Seawater samples were collected using 10 L Niskin bottles attached to the CTD rosette sampler. Nutrients (i.e., ammonium [NH_4_^+^], nitrite + nitrate [NO_2_^**−**^ + NO_3_^**−**^], and phosphate [PO_4_^3**−**^]) were analyzed in the onboard laboratory using a continuous flow auto-analyzer (QuAAtro, Seal Analytical, Germany)^27^. The analytical precision of the nutrient measurements was better than 1%.

In this study, the dissolved inorganic nitrogen (DIN) was defined as the sum of [NH_4_^+^  + NO_2_^**−**^ + NO_3_^**−**^]. Seawater samples for a chlorophyll-*a* analysis were filtered onto 25 mm Whatman GF/F filters, extracted in 90% acetone at 4 °C for 24 h, and quantified using a Turner Designs fluorometer (Trilogy Fluorometer, Turner Designs, USA) with an analytical precision of ± 0.05 μg L^**−**1^^28^. Samples for N_2_O analysis were transferred to 120-mL glass bottles. To inhibit the biological activity, 100 μL of a saturated mercury chloride (HgCl_2_) solution was added to each sample and then sealed with rubber stoppers and aluminum caps^29^. The samples were then stored in the laboratory at ambient ‘laboratory temperature’ (~ 24 °C) until analysis. Wind speeds were observed using a windmill anemometer (05106, RM Young, USA) on the R/V Araon at a height of 30 m (U_30_) above the sea surface, and were then converted from U_30_ to a height of 10 m (U_10_) using a log wind profile method (refer to Supplementary Text S1 and Table S1).

### Dissolved N_2_O measurements using a cavity ring-down spectrometer

For ease and convenience of gas extraction, we used the headspace method to extract dissolved N_2_O gas from the samples (see Supplementary Text S2). Subsamples were obtained by transferring 40 mL water samples from 120-mL glass bottles into a 100-mL glass gas-tight syringe, followed by the addition of 40 mL of high-purity N_2_O-free air. The gas-tight syringe was shaken using an action shaker for 10 min to achieve equilibrium of gases between the sample and headspace phases (Supplementary Fig. S1). This equilibrium gas was injected into a Cavity Ring-Down Spectrometer (CRDS), which is a laser-based technique that uses the optical absorbance characteristics of the gas. CRDS has recently been widely and frequently used to measure greenhouse gases in various marine environments^31−33^. Herein, we used a commercially available CRDS (Model G2308, Picarro Inc., USA) for N_2_O measurements (Supplementary Fig. S1). As N_2_O concentration obtained by the CRDS is the concentration in the headspace, a calculation is required to determine the concentration of dissolved N_2_O in the seawater sample (Eq. 1):

1 $$N_{2} O_{conc.} = \left( {\beta \cdot x \cdot P \cdot V_{w} + \frac{x \cdot P}{{R \cdot T}} \cdot V_{hs} } \right)/V_{w} ,$$

where *β* is the Bunsen solubility (nmol L^**−**1^ atm^**−**1^) determined from the relationship between seawater θ and S^34^; *x* is the dry gas mole fraction (ppb) measured in the headspace; *P* is the atmospheric pressure (atm); *V*_*w*_ is the volume of the water sample (mL); *V*_*hs*_ is the volume of the headspace phase (mL); *R* is the gas constant (0.082057 L atm K^−1^ mol^−1^); and *T* is the equilibration temperature in Kelvin (K)^35^.

To validate the CRDS-based N_2_O measurements, the measurement accuracy was examined by repeatedly measuring an N_2_O standard gas, which was certified as 334.1 ppb by the Korea Research Institute of Standards and Science, before and after the sample measurement with an interval of 20 samples. The measurements of standard gas were well reproduced within a deviation of approximately 3% (Supplementary Fig. S2). In addition, we repeatedly measured the reference water (RW) of known concentration (N_2_O_RW_ = 7.74 nmol L^−1^) obtained by equilibrating the ambient air (N_2_O_air_ = 337.3 ppb) with seawater (T = 20.5 °C and S = 33.93 psu) for 24 h in the laboratory^36^. The N_2_O_RW_ was estimated from the T and S of the equilibrated water^34^. The analytical precision was approximately 4% (Supplementary Table S2).

Because we collected single seawater samples to measure the dissolved N_2_O concentrations during the 2017 summer survey, we conducted measurements of duplicate samples collected at different times and in different environments (Supplementary Fig. S3). The measurement discrepancy between duplicate samples was no greater than 3% (Supplementary Tables S3 and S4).

### Estimations of excess N_2_O and biogeochemical tracers (AOU and N^*^)

The excess N_2_O (ΔN_2_O), which is the amount of biogeochemically derived N_2_O, was estimated as the difference between the equilibrium N_2_O (expressed as [N_2_O]_*eq*_ = N_2_O_*air*_·*β*·*P*, where N_2_O_*air*_ is the atmospheric N_2_O level) and measured N_2_O concentration (N_2_O_*measured*_)^29,37,38^ and is expressed as follows:

2 $${\Delta N}_{2} {\text{O}} \left( {{\text{nmol}}\,{\text{L}}^{ - 1} } \right) = {\text{N}}_{2} {\text{O}}_{measured} - {\text{N}}_{2} {\text{O}}_{eq} .$$

To accurately estimate ΔN_2_O, we need to estimate the time at which a water parcel has its last contact with the atmosphere (i.e., ventilation age) because N_2_O_air_ varies temporally^39^. Although this concept has not been applied in many studies, a few studies have estimated ΔN_2_O by analyzing isotopes^20,25^ or estimating the convection rate^22^. However, in these previous studies, there was also a limitation in that the uncertainty was large for the isotopic composition value or the convection rate in the field was not always constant. Hence, for our calculation, we used sulfur hexafluoride (SF_6_), which displays linear growth over time in the atmosphere (Supplementary Fig. S4). The SF_6_ tracer data were collected during the summer 2015 CLIVAR ARC01 cruise^40^ (Supplementary Fig. S5). The SF_6_-derived ventilation age of a water parcel collected at time *t* was calculated by first converting the measured SF_6_ concentration (femtomoles kg^−1^) into its partial pressure (*p*SF_6_), based on the θ and S of the sample. The *p*SF_6_ value was then matched to the atmospheric growth record for SF_6_ to determine the calendar year (τ) in which the SF_6_ concentration in the water sample would have been in equilibrium with the atmosphere (i.e., the “ventilation date”)^41^. The *p*SF_6_ age or “ventilation age” of a water parcel is given as *t* − τ (Fine, 2011). The N_2_O_air_ history affecting the WAO water column was based on the SF_6_-derived calendar age (Supplementary Table S5 and Fig. S6).

The relationship between ΔN_2_O and other biogeochemical tracers, such as the apparent oxygen utilization (AOU = [*DO*]_*eq* (*θ,S*)_ – [*DO*]_*measured*_) and N^*^ (= [NO_3_^*−*^]_*measured*_ − *R*_*N:P*_ × [PO_4_^3−^]_*measured*_, where *R*_*N:P*_ is the Redfield ratio) has been widely used to estimate the biogeochemical production and/or consumption of N_2_O in various marine environments^19,37,38,42−46^. AOU is typically interpreted as the amount of DO consumed during remineralization^47,48^, a positive linear relationship between ΔN_2_O and AOU indicates that nitrification (NH_4_^+^  → NO_2_^−^ → NO_3_^−^) is the main pathway of ΔN_2_O production^29,44^. In addition, N^*^ has been widely used as an indicator of excess nitrogen (e.g., a nitrogen fixation) or deficit (e.g., denitrification: NO_3_^−^ → NO_2_^−^ → N_2_O/N_2_) relative to phosphorus^49^. In this study, N^*^ was calculated as [DIN] − 16 × [PO_4_^3−^], where 16 is the Redfield ratio of N to P. A negative linear relationship between ΔN_2_O and N^*^ indicates that ΔN_2_O is mainly produced through denitrification^13,46,50^.

## Results and discussion

### Summer oceanographic conditions

During the summer open-water season, it is apparent that the physical and biogeochemical properties of the WAO surface waters show a latitudinal gradient from the Bering Strait to the Chukchi Borderland^12^. The southern Chukchi (SC) (i.e., Bering Strait to Chukchi Shelf), which is mainly influenced by relatively warm and nutrient-enriched Pacific waters, is a highly biologically productive marine environment displaying a generally high chlorophyll-*a* biomass^12,51^ (Fig. 1a). By contrast, the northern Chukchi (NC) (i.e., the Chukchi Borderland and Canada Basin) is primarily driven by freshwater inputs from melting sea ice and rivers, and is characterized as cold, fresh, and oligotrophic, displaying a generally low chlorophyll-*a* biomass (Fig. 1a)^52−54^.

To investigate the hydrographic conditions during the summer of 2017 in the WAO, we analyzed the vertical distributions of θ, S, DO, and DIN along a latitudinal transect from the Bering Strait to the Chukchi Borderland (Fig. 1a–c,e,f) and used a θ–S diagram to examine the composition of water masses (Fig. 1d). The distributions of θ, S, and DIN in the surface waters (< 50 m depth) suggest generally warmer, more saline, and DIN-richer waters in the SC (mean θ_0–50 m_ of 5.14 °C, S_0–50 m_ of 32.33 psu, and DIN_0–50 m_ of 5.31 μmol L^−1^) compared to the NC (mean θ_0–50 m_ of − 1.00 °C, S_0–50 m_ of 29.65 psu, and DIN_0–50 m_ of 1.20 μmol L^−1^) (Fig. 1b,c,f). In addition, the DIN_0–10 m_ inventory is higher in the SC (mean of 0.61 g m^−2^) than in the NC (mean of 0.01 mg m^−2^) (Fig. 1f), indicating a greater potential for driving a higher primary production in the SC, as found by Grebmeier et al.^55^. Meanwhile, the distribution of DO exhibited the opposite behavior (mean DO_SC(0–50 m)_ of 333.8 μmol L^−1^ and DO_NC(0–50 m)_ of 385.4 μmol L^−1^) (Fig. 1e). Based on the geographical distribution, as shown in the θ-S diagram (Fig. 1d), two different water masses are likely to be involved in the mixing process within the surface waters of the study area: warm, saline, and nutrient-enriched SC waters (i.e., Pacific Summer Water (PSW), which is also called the Bering Summer Water)^56−59^ and cold, fresh, and nutrient-depleted NC waters (herein referred to as freshwater (FW))^56,57,60,61^. Owing to the distinct physicochemical contrast between mixing PSW and FW, a frontal zone (FZ) arises between them (located between St. 9 and St. 10 (~ 73° N), Fig. 1)^62^.

The intermediate depths (50**–**200 m) in the NC are completely occupied with cold (< 0 °C), saline (31.5–33.6 psu), DO minimum (~ 170 μmol L^−1^), and nutrient-rich (DIN > 10 μmol L^−1^) waters. These water characteristics are associated with Pacific Winter Water (PWW)^57,58,60,61^, which forms in the Bering Sea during winter. The PWW is identified by a signature minimum N^*^ on the θ-S diagram (Fig. 1d).

Below the PWW, the maximum θ (~ 1.25 °C) and high salinity (~ 34.89 psu) water observed between 200 and 1000 m in the deep NC (Fig. 1b,c) is typically called Atlantic Water (AW)^56,58,60^. In contrast to PWW, AW is associated with a relative maximum N^*^ (Fig. 1d). The densest waters (θ < 0 °C and S =  ~ 34.95 psu), with a relatively uniform θ/S, are distributed from below ~ 1000 m to the bottom and are defined as Arctic Bottom Water (ABW)^61,63^. ABW is associated with a maximum N^*^ signature along the transect (Fig. 1d).

**Figure 2:**
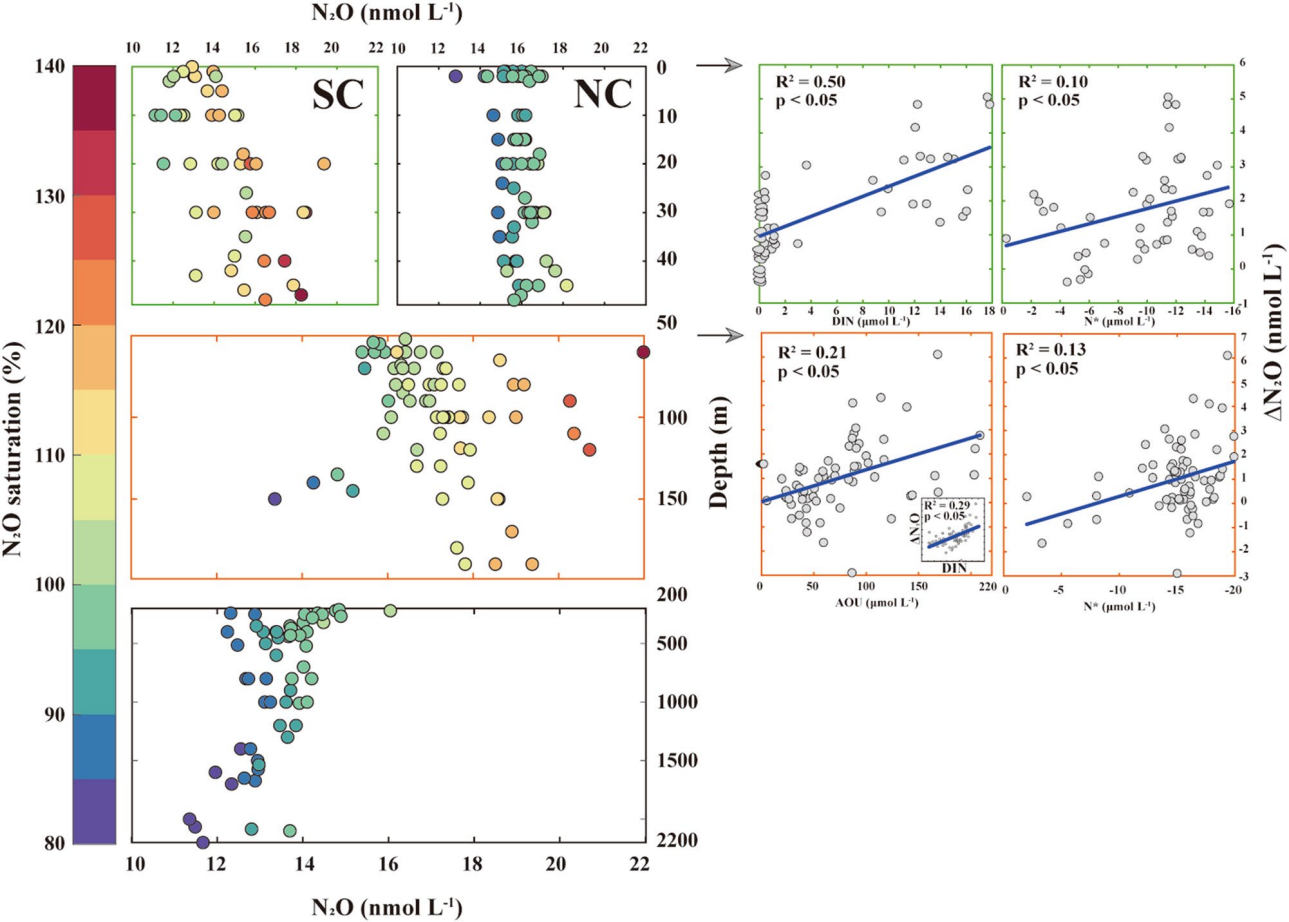
Vertical distribution of N_2_O measured in the WAO water column during the summer of 2017 with saturation levels (N_2_O_sat_ (%) = (N_2_O_measured_/N_2_O_eq_) $\times$ 100; blue to red gradient color bar). Based on the vertical composition of water masses in the study area (surface, Pacific summer water and fresh water; intermediate, Pacific Winter Water; and deep areas, Atlantic Water and Arctic Bottom Water), we represent the vertical N_2_O distribution by dividing into three layers (i.e., surface, 0–50 m; intermediate, 50–200 m; and deep layer, 200–2200 m). To show contrasting values associated with saturation levels (%), the surface layer is shown separately for SC and NC. To investigate the potential sources of biogeochemically derived N_2_O (ΔN_2_O) production, the figures on the right-hand side show the correlations of ΔN_2_O with DIN and N^*^ in the surface layer of the SC (green color boxes), and with AOU and N^*^ in the intermediate layer (orange color boxes), including statistical information (R^2^ and *p* values). The inset shows the correlation between ΔN_2_O and DIN in the intermediate layer. Note that this figure was generated using MATLAB program (ver. R2019b and www.mathworks.com).

In summary, during the summer of 2017, the study area consisted of five water masses: PSW, FW, PWW, AW, and ABW, recognizable in both the vertical and horizontal directions. Further details on the physicochemical characteristics of these water masses are provided in Supplementary Table S6. To discuss N_2_O dynamics, the water column was divided into three layers based on the vertical distribution of the water masses in the study area: surface (0–50 m), intermediate (50–200 m), and deep (200–2200 m) areas (Fig. 2).

### N_2_O dynamics: distribution and controlling factors

Within the surface layer, the vertical distribution of SC N_2_O concentrations shows a pattern of increasing concentration with depth (i.e., surface, ~ 11.1 nmol L^−1^; up to 50 m, ~ 19.4 nmol L^−1^), whereas those in the NC are uniformly distributed with values of approximately 16 nmol L^−1^. The mean N_2_O concentrations of the SC and NC are estimated as 14.7 ± 2.1 and 15.9 ± 0.8 nmol L^−1^, respectively; however, the mean N_2_O saturation is higher in the SC (113% ± 10%, over-saturation) than in the NC (95% ± 5%, under-saturation).

Toyoda et al.^25^ published N_2_O measurements taken in 2014 and 2015. In the 2014 data vertical N_2_O concentrations in the SC were observed to increase with depth (up to 23 nmol kg^−1^), while those in the 2015 data were homogeneous (~ 15 nmol kg^−1^) throughout the water column. Toyoda et al. surmised that the annual variations in N_2_O distribution may be a function of whether or not stratification occurs within the water column. Based on our results, we suggest that waters over-saturated with N_2_O in the SC bottom water could migrate towards the surface as a result of vigorous vertical mixing.

There is a subsurface N_2_O maximum (~ 22 nmol L^−1^) in the intermediate layer with a mean N_2_O concentration and saturation of 17.2 ± 1.5 nmol L^−1^ and 107% ± 10%, respectively. PWW, showing the lowest DO (~ 170 μmol L^−1^), nutrient-rich (DIN > 10 μmol L^−1^), and relatively minimum N^*^ (− 19.91 μmol L^−1^) features, fully occupies the intermediate layer (Fig. 1). Consequently, the highest N_2_O concentration (22.0 nmol L^−1^) and saturation (138%) are found in the intermediate layer. These results are consistent with those of previous studies^13,19,21,25^ where subsurface N_2_O maxima ranged from 18.1 to 24.6 nmol L^−1^ and were recorded at depths of between 100 and 200 m.

The deep layer is mainly composed of AW (generally distributed between 200 and 1000 m with a mean SF_6_-based ventilation age of 24.3 ± 3.9 years) and ABW (generally, below ~ 1000 m with an SF_6_-based ventilation age of 46.6 ± 14.5 years) (Supplementary Fig. S6). Under these conditions of great age and relative stability, N_2_O concentrations should show little variation (Supplementary Fig. S7 and Supplementary Table S7). The N_2_O concentrations were constant at 13.9 ± 1.0 nmol L^−1^ in the AW zone, and showed a slightly decreasing trend in the ABW zone (bottom, 12.9 ± 0.8 nmol L^−1^). The saturation values in both zones are mostly less than 100% (i.e., under saturated conditions).

The distribution of SC N_2_O exhibited different patterns from the distribution of NC N_2_O within the surface layer. These results are indicators of the effect of the physical solubility, which is mainly determined by T and S^34^, and is dominant in the NC (cold and fresh) compared to the SC (warm and saline)^23^. In addition to the physical solubility, Randall et al.^64^ reported that the N_2_O of sea-ice meltwater was greatly under-saturated, and several studies^13,19,21,24^ have suggested that the under-saturated N_2_O in the NC surface water may be related to the dilution of melting sea ice.

The over-saturated SC, which is known to be a high biological productivity region^51^, is presumed to have biogeochemically derived N_2_O (i.e., ΔN_2_O) production that also contributes to the concentration. In addition, the ΔN_2_O production of each water parcel was precisely calculated, resulting from the SF_6_-derived ventilation date. To identify potential ΔN_2_O production sources in the SC, we evaluated both the negative linear relationship between ΔN_2_O and N^*^ and the positive linear relationship between ΔN_2_O and DIN. The relationship between ΔN_2_O and AOU was not considered. This approach was taken because the SC is a shallow shelf region where the entire water column is kept in relatively close contact with the atmosphere. Plots of ΔN_2_O versus DIN show significantly positive correlations (R^2^ = 0.50, *p* < 0.05) (Fig. 2), suggesting that nitrification is likely to serve as the main sources of ΔN_2_O in the SC^65,66^. Interestingly, plots of ΔN_2_O versus N^*^ show weak negative correlations (R^2^ = 0.10, *p* < 0.05) and scattered distributions. It has been suggested by both Hirota et al.^20^ and Toyoda et al.^25^ that ΔN_2_O production in the SC can be attributed to sedimentary denitrification resulting from the coupling of the inverse correlation between ΔN_2_O and N^*^ and the stable isotope composition of N_2_O. Here, the somewhat ‘scattered’ relationship observed between ΔN_2_O and N^*^ may be due to the efflux of N_2_O produced by sedimentary denitrification. Regrettably this hypothesis remains un-tested due to the absence of robust sedimentary data, leaving scope for future work.

Similar results have been reported in earlier studies. According to Zhang et al.^13^, the N_2_O concentration in the SC increased with depth, and both the ΔN_2_O-AOU and ΔN_2_O-N^*^ relations were significant, suggesting that vigorous ΔN_2_O production is generated through sedimentary nitrification and denitrification. Wu et al.^21^ also observed high N_2_O concentrations corresponding simultaneously to the oxygen minimum and high concentrations of NH_4_^+^ over the Chukchi Sea continental shelf, and suggested that N_2_O production is derived from sedimentary nitrification, denitrification, and nitrification in the water column. Fenwick et al.^19^ also suggested that the significant relationship between ΔN_2_O and N^*^ represents the primary source of denitrification and that the significant scatter found in this relationship is due to the influence of other nitrogen cycling processes on ΔN_2_O production, albeit an insignificant relationship between ΔN_2_O and AOU. The results of these studies support our findings.

The low temperatures (i.e., high gas solubility) characteristic of PWW may be a potential cause of the N_2_O maximum observed within the intermediate layer. If, however, N_2_O concentrations were high solely due to the water parcel’s high solute capacity, we should expect dissolved DO concentrations to be similarly elevated. However, the DO concentrations measured within the intermediate layer were low and the AOU was high. SF_6_ is likewise affected by solubility as other gases. Based on the assumption that dissolved SF_6_ concentrations in any given water parcel will be in equilibrium with the adjacent atmosphere prior to being ventilated, one can determine the ventilation date and the precise equilibrium N_2_O concentrations of the water parcels corresponding to that ventilation date through reference to the SF_6_ concentrations. Compared to the equilibrium N_2_O levels at the time of water mass formation (i.e., ventilation date), the N_2_O concentrations observed in PWW were clearly indicative of over-saturation.

The highest N_2_O concentration and saturation in the intermediate layer suggest that here N_2_O production is significant. The relationships between ΔN_2_O and AOU have positive (R^2^ = 0.21, *p* < 0.05) correlations. In addition, a positive linear relationship between ΔN_2_O and DIN is well represented (R^2^ = 0.29, *p* < 0.05). The results suggest that nitrification may contribute to ΔN_2_O production in the intermediate layer. Meanwhile, the relationships observed between ΔN_2_O and N^*^ were weak (R^2^ = 0.13, *p* < 0.05) potentially as a result of interaction between the bottom water and sediments on the shelf slope.

In addition, the lateral input of shelf waters (i.e., PSW) might contribute to the N_2_O concentrations of the intermediate layer^20^. Zhang et al.^13^, Wu et al.^21^, and Toyoda et al.^25^ have all suggested that the subsurface N_2_O maximum may be attributable not only to its local production within the water column (e.g., nitrification), but also to its northward transportation from the SC. Given that PSW input increases with latitude^10^, lateral transport of N_2_O may be a significant factor in determining the characteristics of surface/intermediate layers within the NC.

According to Zhan et al.^22^ and Fenwick et al.^19^, in the deep layer of the Arctic Ocean, both the decreasing N_2_O and oxygen with depth and the estimated NO_3_^−^ regeneration rate (2.3 × 10^−5^ μmol L^−1^ a^−1^) indicate that nitrification may be insignificant for N_2_O accumulation. Denitrification may also be insignificant for N_2_O accumulation because of the relatively high oxygen concentration. It has been suggested that the N_2_O levels observed in the deep layer samples may have occurred because the water was last ventilated during pre-industrial times. This hypothesis is based on the estimated convection rate. Offering their own interpretation of the isotopic data, Toyoda et al.^25^ suggested that the N_2_O concentrations observed in the deep layer were derived from a mixture of water ventilated under pre-industrial atmospheric conditions and N_2_O produced by nitrification occurring within the water column.

Here, based on the SF_6_-based ventilation age, ventilation dates of the deep water masses (i.e., AW and ABW) were determined to be from circa 1955.9 to 1995.1. The N_2_O concentrations of the deep layer were under-saturated, compared to equilibrium values in atmospheric N_2_O of the ventilation dates. These uniformly under-saturated N_2_O concentrations and the relatively homogeneous hydrographic properties suggest that deep N_2_O concentrations are mainly determined by physical mixing processes such as advection and formation, rather than the involvement of biogeochemical processes (i.e., nitrification and denitrification).

### Estimation of N_2_O flux: WAO source or sink during the summer of 2017?

To determine whether the WAO was a net source or sink for atmospheric N_2_O during the summer of 2017, we used the air-sea gas exchange equation to estimate the N_2_O flux as follows:

3 $${\text{N}}_{2} {\text{O}} flux = { }k_{w} \cdot \left( {\left[ {{\text{N}}_{2} {\text{O}}} \right]_{measured}^{surface} - \left[ {{\text{N}}_{2} {\text{O}}} \right]_{eq} } \right),$$

where ${\left[{\mathrm{N}}_{2}\mathrm{O}\right]}_{\mathrm{measured}}^{\mathrm{surface}}$ is the surface-water N_2_O concentration, and *k*_*w*_ is the gas transfer velocity (cm h^−1^). In previous studies^19−21,23^, three model approaches were used to estimate *k*_*w*_ in the WAO, i.e., those developed by Wanninkhof^67^, Wanninkhof and McGillis^68^, and Nightingale et al.^69^. It should be noted that we used the *k*_*w*_ of Wanninkhof^70^ instead of that of Wanninkhof^67^ to more accurately reflect *k*_*w*_ in Eq. (3) (refer to Supplementary Text S1). Fenwick et al.^19^ and Zhan et al.^24^ used the weighted mean wind data (60 days prior to sampling) to avoid an overestimation of the instantaneous wind data in the process of calculating *k*_*w*_. However, we used the mean wind data during sampling to provide results as observation-based snapshots. The mean differences in the estimated N_2_O flux from the three models are 0.3 μmol N_2_O m^−2^ day^−1^ in the SC and 0.2 μmol N_2_O m^−2^ day^−1^ in the NC (Supplementary Table S8). To facilitate the presentation of our results, we employed the mean value of the N_2_O flux averaged from the three models.

**Figure 3:**
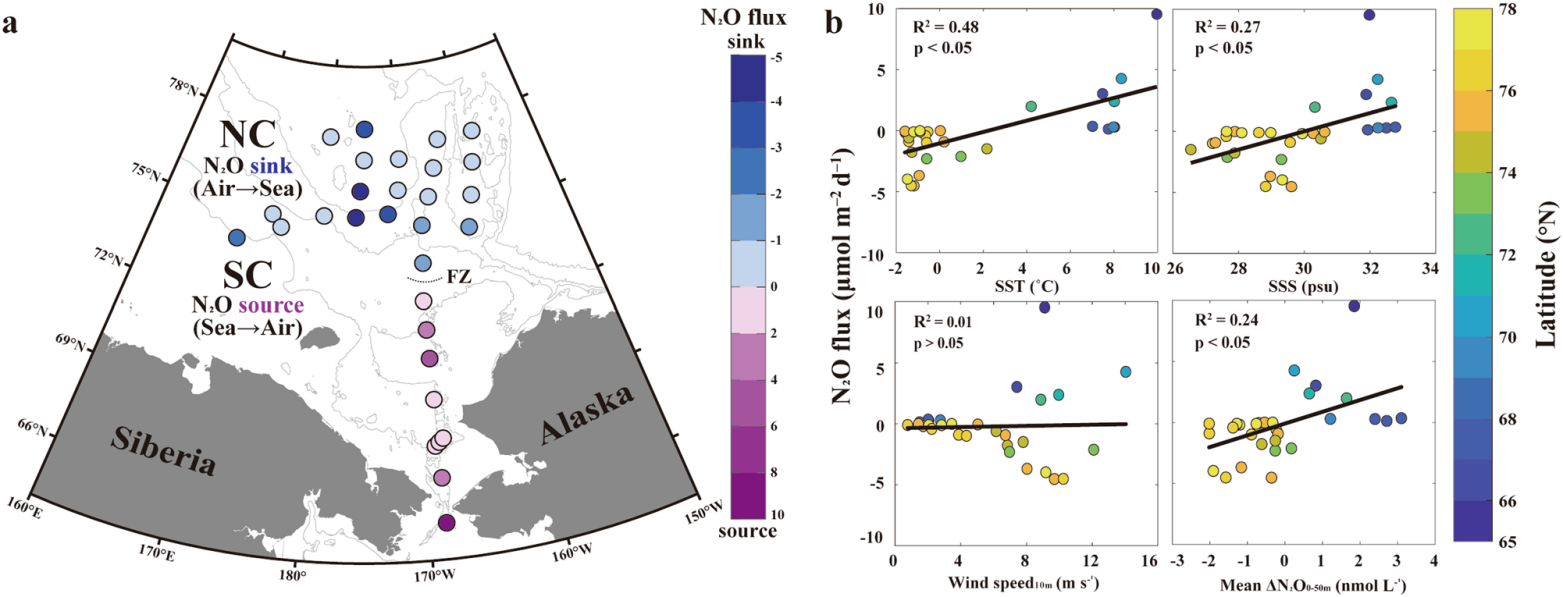
(**a**) Spatial distribution of mean WAO surface N_2_O fluxes across the air–sea interface during the summer of 2017 (blue to purple gradient color bar; sink (−), air → sea, and source (+), sea → air) and (**b**) correlations of N_2_O flux with SST, SSS, wind speed at 10 m (U_10_), and mean ∆N_2_O_0–50 m_ (averaged between surface and 50 m), including statistical information (R^2^ and *p* values). The color bar represents the latitude. Note that this figure was generated using MATLAB program (ver. R2019b and www.mathworks.com).

A map illustrating the spatial distribution of the summer WAO N_2_O fluxes (Fig. 3a) indicates that the SC (Sts. 1**–**9) is exclusively occupied by positive (+ : sea → air) N_2_O fluxes ranging from 0.1 to 8.6 μmol N_2_O m^−2^ day^−1^, whereas the NC (Sts. 10**–**30) is dominated by negative (‒: air → sea) N_2_O fluxes ranging from − 4.3 μmol N_2_O m^−2^ day^−1^ to zero. Interestingly, a contrasting distribution of the N_2_O fluxes between the SC (+) and NC (−) is apparent along the FZ, similar to that suggested for the physical and biogeochemical properties determined by Lee et al.^12^ (see their Fig. 1).

Our results agree well with those of several previous studies. The positive N_2_O fluxes were estimated by Hirota et al.^20^, although their research area was limited to the south of the SC, and the investigation was conducted in few locations. Wu et al.^21^ and Zhan et al.^23^ likewise estimated positive N_2_O fluxes in the SC and negative N_2_O fluxes in the NC, although the flux values were calculated using only one model. In addition, Fenwick et al.^19^ estimated relatively lower fluxes, suggesting that these results may be due to either of the different calculation approaches (e.g., weighted mean wind data over 60 days prior to sampling), varying oceanographic conditions (e.g., dilution by melting of sea ice with low N_2_O concentration) or both. In addition, the locations are intensive near the coast. Zhan et al.^24^ also used the weighted mean wind data for three different models, and the SC was identified as a source of atmospheric N_2_O, but the NC was not identified as either a source or a sink. Despite the similar surface N_2_O concentrations (~ 16.5 nmol L^−1^) with our dataset, these different results may be due to different calculation approaches (i.e., different air-sea exchange models and mean wind data). Toyoda et al.^25^ estimated negative and positive N_2_O fluxes in the SC, respectively, in 2014 and 2015. However, the fluxes investigated during late summer were more positive than that during early autumn in each of two years. They suggested that the SC can act as both source and sink depending on the season.

Until this study, there has not been a clear estimation of the controlling factors of N_2_O fluxes in the WAO. To investigate the factors controlling the WAO N_2_O fluxes of summer 2017 for the first time, we examined correlations between the N_2_O flux and physical and biogeochemical parameters, such as the sea surface temperature (SST), sea surface salinity (SSS), wind speed at 10 m (U_10_), and mean ∆N_2_O_0–50 m_ (averaged between surface and 50 m) (Fig. 3b). The results showed that the relationships between the N_2_O flux and the SST (R^2^ = 0.48, *p* < 0.05), SSS (R^2^ = 0.27, *p* < 0.05), and mean ΔN_2_O (R^2^ = 0.24, *p* < 0.05) are significant (Fig. 3b), whereas the correlation with U_10_ is not.

Taken together, these results suggest that during the summer of 2017, the SC acted as a source (mean of + 2.3 ± 2.7 μmol N_2_O m^−2^ day^−1^) and the NC served as a sink (mean of − 1.3 ± 1.5 μmol N_2_O m^−2^ day^−1^) for atmospheric N_2_O. The summer WAO N_2_O fluxes were significantly influenced by physical variables associated with the solubility (i.e., SST and SSS) and biogeochemically derived N_2_O production, implying that the distribution of the WAO N_2_O flux is typically strongly susceptible to environmental changes.

**Figure 4:**
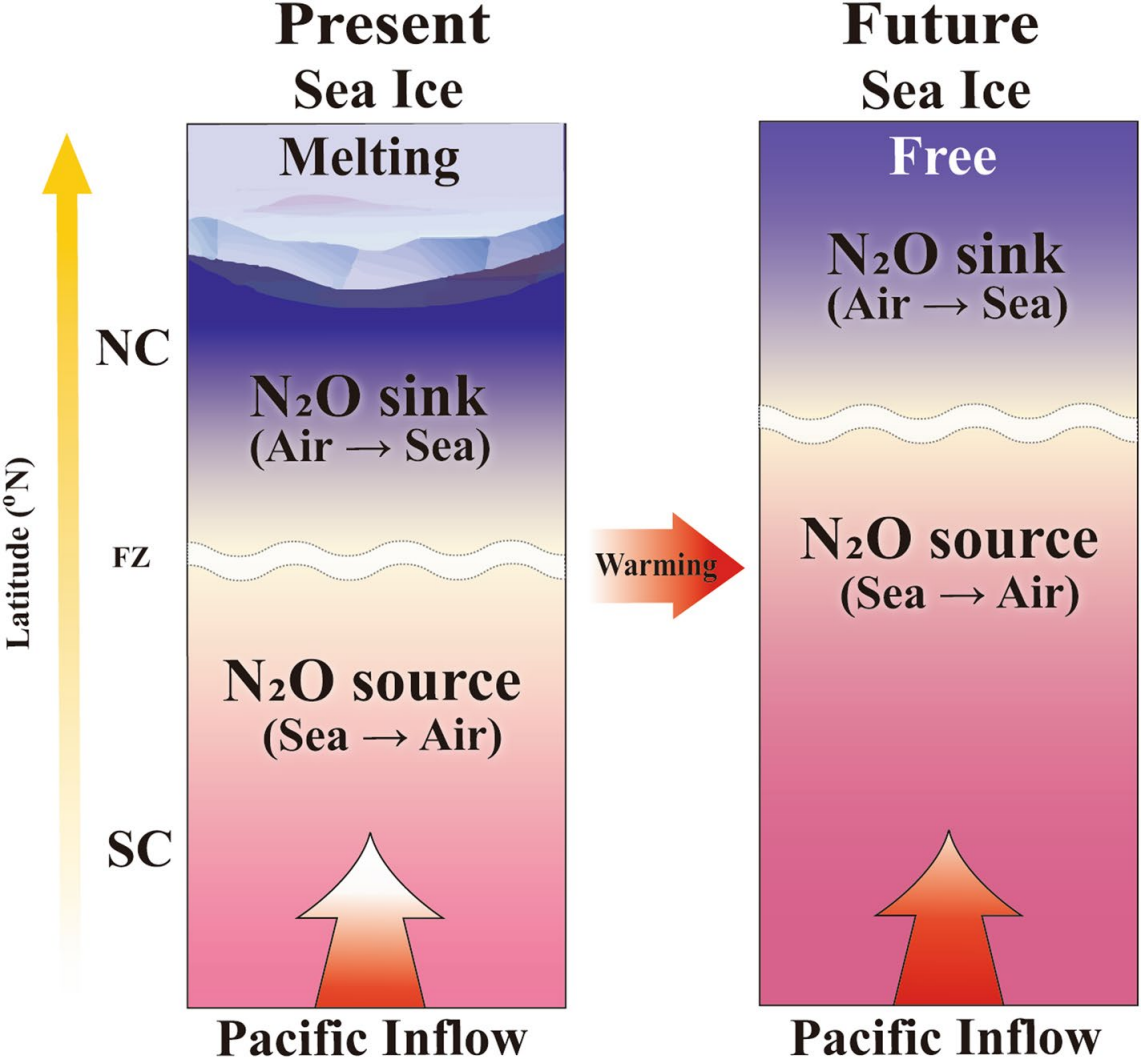
Illustration showing future changes in the distribution of the WAO N_2_O flux constrained by the positive feedback scenario of increasing inflow of Pacific waters and rapidly declining sea-ice extent under accelerating Arctic warming. Note that this figure was generated using Adobe Illustrator CC program (ver. 2018 and www.adobe.com).

A multitude of environmental changes that occur in the WAO may directly and indirectly influence the distribution of WAO N_2_O fluxes (see Fig. 3a). Among the changes observed, the increasing inflow of the Pacific waters^10,72−73^ and the rapidly declining sea-ice extent^9,11,74,75^ have received substantial attention to date. Based on these two phenomena, we can speculate that the distribution of WAO N_2_O fluxes revealed in this study will change in the future (Fig. 4). Lewis et al.^76^ suggested that the increased phytoplankton biomass sustained by an influx of new nutrients, in addition to sea-ice reduction, has driven the Arctic Ocean (e.g., Chukchi Sea) to be increasingly more productive. The increased biomass would lead to intense nitrification and potentially benthic denitrification, resulting in increased N_2_O production within the water column. The increasing inflow of warm and nutrient-enriched Pacific waters into the WAO would likely extend the productive SC region northward, leading to an enlarged WAO role as an N_2_O source (positive, sea → air), whereas a rapid loss of the sea ice extent may ultimately lead to a sea-ice-free NC with a northward shift, resulting in a diminished role as an N_2_O sink (negative, air → sea). Should this potential scenario come to pass, we would expect the WAO to become an oceanic N_2_O “hot spot” source region, and we therefore suggest that this positive feedback scenario should be considered in an effort to improve the future trajectory of WAO changes.

## Summary and conclusions

We investigated the distributions of the N_2_O concentration and flux, their controlling factors, and the role of the WAO as a source or sink for atmospheric N_2_O during the summer of 2017. In the surface layer (0–50 m, consisting of PSW + FW), the mean N_2_O concentration of the SC and NC is estimated to be 14.7 ± 2.1 and 15.9 ± 0.8 nmol L^−1^, respectively. However, the mean N_2_O saturation was higher in the SC (113% ± 10%, over-saturation) than in the NC (95% ± 5%, under-saturation). This result indicates that the effect of the physical solubility is dominant in the NC (cold and fresh) compared to the SC (warm and saline), and that the over-saturated SC is likely to gain additional biogeochemically derived N_2_O (i.e., ΔN_2_O) production through nitrification. The intermediate layer (50–200 m, occupied by PWW) exhibits a subsurface N_2_O maximum (> 20 nmol L^−1^) with a linear relationship between ΔN_2_O and AOU (positive). In the deep layer (200–2200 m, consisting of AW and ABW), deep N_2_O concentrations are mainly determined by the physical mixing processes, such as advection and formation. The estimated mean N_2_O flux across the air–sea interface was + 2.3 ± 2.7 μmol N_2_O m^−2^ day^−1^ in the SC region (i.e., source) and − 1.3 ± 1.5 μmol N_2_O m^−2^ day^−1^ in the NC region (i.e., sink), respectively, showing a contrasting distribution of N_2_O flux.

As our study was based on a single investigation, it is impossible for us to represent the entire 2017 calendar year, or even the entire summer of 2017. We are not, however, alone in suffering from this ‘limited data’ impediment. The Arctic Ocean is an extreme environment, acquiring year-round data is very difficult and extremely costly. Consequently, our results are a mere snapshot of what is undoubtedly a much bigger picture. We intend to propose a direction for future work based on our experience of undertaking this study.

If Arctic changes are accelerated and consequently drive the Arctic Ocean in a more productive manner, the WAO may become an oceanic N_2_O “hot spot” source region. Given that these processes are relevant to global climate change, additional observations of the time series and more open-water seasons are required to support this scenario. Therefore, attention should be paid to future N_2_O dynamics in the WAO.

## Supplementary Information

Supplementary Information.

## Data Availability

Hydrographic data are available in Korea Arctic Ocean-data System (http://kaos.kopri.re.kr/cmm/main/mainPage.do). Atmospheric N_2_O data are available in ESRL (https://www.esrl.noaa.gov). The SF_6_ data collected from the 2015 CLIVAR ARC01 cruise are available in CCHDO (https://cchdo.ucsd.edu/). The N_2_O flux and wind data are presented in the Supporting Information.
