# Supplementary material for: N2O dynamics in the western Arctic Ocean during the summer of 2017
Source: Sci Rep. 2021 Jun 15;11:12589. doi: 10.1038/s41598-021-92009-1 (PMC8206351; doi:10.1038/s41598-021-92009-1)
Supplement: Supplementary file 1 — Supplementary Information. [file 41598_2021_92009_MOESM1_ESM.docx]

**-Supplementary Information-**

**N_2_O Dynamics in the Western Arctic Ocean during the Summer of 2017**

**Jang-Mu Heo^1^, Seong-Su Kim^1^, Sung-Ho Kang^2^, Eun Jin Yang^2^, Ki-Tae Park^2^,**

**Jinyoung Jung^2^, Kyoung-Ho Cho^2^, Ju-Hyoung Kim^3^, Alison M. Macdonald^4^,**

**Joo-Eun Yoon^1^, Hyo-Ryeon Kim^1^, Sang-Min Eom^1^, Jae-Hyun Lim^5^,**

**and Il-Nam Kim^1*^**

^1^ Department of Marine Science, Incheon National University, Incheon 22012, South Korea.

^2^ Korea Polar Research Institute, Incheon 21990, South Korea.

^3^Faculty of Marine Applied Biosciences, Kunsan National University, Gunsan 54150, South Korea

^4^Woods Hole Oceanographic Institution, MS 21, 266 Woods Hold Rd., Woods Hole, MA 02543, USA.

^5^ Fisheries Resources and Environmental Research Division, East Sea Fisheries Research Institute, National Institute of Fisheries Science, Gangneung 25435, South Korea.

***Correspondence: Il-Nam Kim (ilnamkim@inu.ac.kr)**

**Contents of this file:**

Text S1 to S2

Figures S1 to S6

Tables S1 to S6

**Text S1. Estimation of gas transfer velocity**

Three models have commonly been used to estimate the *k_w_* in the western Arctic Ocean (WAO) (Wanninkhof (1992)[^1^](#_ENREF_1), Wanninkhof and McGillis (1999; WM1999)[^2^](#_ENREF_2); Nightingale et al. (2000; N2000)[^3^](#_ENREF_3)). We used Wanninkhof (2014; W2014)[^4^](#_ENREF_4) rather than Wanninkhof (1992)[^1^](#_ENREF_1) as it provides a more accurate *k_w_* in the N_2_O flux estimation. The *k_w_* equations are given as:

$k_{w}^{WM1999}=0.0283\times U_{10}^{3}\times\left( \mathrm{Sc}/{660} \right)^{-0.5}$, (S1)

$k_{w}^{N2000}=\left( 0.333\cdot U_{10}+0.222\cdot U_{10}^{2} \right)\times\left( \mathrm{Sc}/{600} \right)^{-0.5}$, (S2)

$k_{w}^{W2014}=0.251\times U_{10}^{2}\times\left( \mathrm{Sc}/{660} \right)^{-0.5}$, (S3)

The three *k_w_* models use the wind speed at 10 m above the sea surface (U_10_) and the Schmidt number (*Sc*). *Sc* is estimated as the kinematic viscosity of seawater divided by the diffusion coefficient of N_2_O in seawater[^5^](#_ENREF_5)^,^[^6^](#_ENREF_6).

During the summer 2017 cruise, wind speeds were observed using a windmill anemometer (05106, RM Young, USA) at a height of 30 m above the sea surface on the R/V Araon. To convert mean wind speed from 30 m height (U_30_) to a 10 m height (U_10_), we used a log wind profile method as follows[^7^](#_ENREF_7):

$U_{10}=U_{30}\times\frac{\ln\left( {{10}_{m}-Z_{h}}/{Z_{0}} \right)}{\ln\left( {{30}_{m}-Z_{h}}/{Z_{0}} \right)}$, (S4)

where ln is the natural logarithm and *Z*$₀$ is the roughness length (m) determined by the size and distribution of the roughness elements it contains. We used an open ocean *Z_0_* value of 0.0001 m[^8^](#_ENREF_8) (Supplementary Table S1). More generally, Equation (S4) includes a zero-plane displacement (*Z_h_*), the height in meters above the ground at which wind speed goes to zero as a result of flow obstacles such as trees or buildings. However, there are no such obstacles over the open ocean so, *Z_h_* is omitted from the calculation.

**Text** **S2. Information about the CRDS-based dissolved N_2_O measurement system**

Gas chromatography (GC) is the most widely used analytical method for N_2_O measurements[^9^](#_ENREF_9). However, high-performance equipment, such as the cavity ring-down spectrometer (CRDS), has been developed and recently become commercially available, as it requires less material volume, shorter measurement time, and a more friendly manageable system than the GC system[^5^](#_ENREF_5)^,^[^10^](#_ENREF_10).

The principle of CRDS is optical spectroscopy to quantify the N_2_O concentration in the gas phase. Traditional optical spectroscopy determines the concentration through the measurement of absolute absorbance. In many cases, the basic characteristics of these spectrometers reduce the sensitivity, affecting accuracy owing to limitations such as light source fluctuation, interference fringes, and path length of light[^5^](#_ENREF_5). Meanwhile, the CRDS determines the concentration through the optical signal’s (i.e., light) rate of decay, allowing for more accurate and stable measurements.

The CRDS contains an optical cavity with three mirrors. When the measurement begins, light from a single-frequency laser enters the cavity and is reflected on the mirrors. After the laser is shut off, most of the light remains trapped within the cavity for a long period of time, producing an effective path length of tens of kilometers through the sample[^11^](#_ENREF_11). The long path length of the system allows the high-resolution detection of N_2_O according to the Lambert–Beer Law. CRDS was designed to maintain the optimal temperature (45 °C) and pressure (140 torr) during the measurement to minimize the dispersion of the spectrum. This allows a precise separation of the wavelength affected by the N_2_O concentration. Although the algorithm for correction of humidity automatically calibrates the error that is affected by humidity under 2 ppm, moisture interrupts the laser in its measurement of the extracted N_2_O gas. Accordingly, we used an absorbent (drierite) to reduce the moisture of the equilibrated gas.

In general, CRDS is used for continuous measurement, requiring a large amount of gas for quantification. However, a small amount of gas was extracted using our headspace method, and the flow restrictor and small isotope inlet module (SSIM) were added to the CRDS. The flow rate decreased from 250 to 25 mL min^-1^. However, the relatively long distance that the gas must travel on its way into the cavity when using the SSIM could result in the partial loss of the gas. To mitigate any possible effects that might arise due to this issue, system pressure was monitored throughout the procedure and was rechecked for potential leakage during sample measurement. An N_2_O standard gas was also measured continuously to ensure the stability of the CRDS system (Supplementary Fig. S2). Moreover, the apparatus, including the cavity itself, was thoroughly washed using N_2_O-zero air prior to the injection of each sample.

The optical signal obtained by the detector is used to measure the light’s rate of decay. This rate of decay is mathematically described as follows:

$I_{t}=I_{t0}exp\left[ \frac{-t}{\tau} \right]$, (S5)

$\alpha= \frac{1}{c\tau}$, (S6)

where *I* is the light intensity, *c* is the speed of light, *τ* is the time required for the light to completely decay within the cavity (cavity ring-down time), and *α* is the cavity gas loss per unit length (ppm cm^-1^). In constant temperature and pressure conditions, gas concentration is proportional to the rate of decay. Using this relationship, the CRDS system calculates the gas concentration in mole fraction (ppm).

The CRDS system requires no calibration prior to use because it calculates the concentration of a target gas using only two variables: the optical signal’s rate of decay and the speed of light. An alternative approach for precisely measuring the aforementioned rate of decay would be to employ a laser (i.e., a light source) with an appropriate wavelength. The CRDS is equipped with a system designed to monitor and regulate the wavelength of the laser and ensure that the laser is properly tuned before measurements are taken.

After the tuning has been completed, the CRDS initiates the process of measuring the samples. Because these measurements are carried out continuously, the N_2_O values gradually increase from zero after gas (equilibrated to the seawater sample) are placed into the cavity. This zero value is the result of initial system washing, which is performed using air devoid of N_2_O. After sample injection and the stabilization of the cavity environment, a mean N_2_O value is determined.

Recently, CRDS has been widely and frequently used to measure greenhouse gases in various marine environments[^11-15^](#_ENREF_11). In this study, we measured dissolved N_2_O concentrations from the samples using the CRDS system (G2308, PICARRO Co., USA) (Supplementary Figure S1).


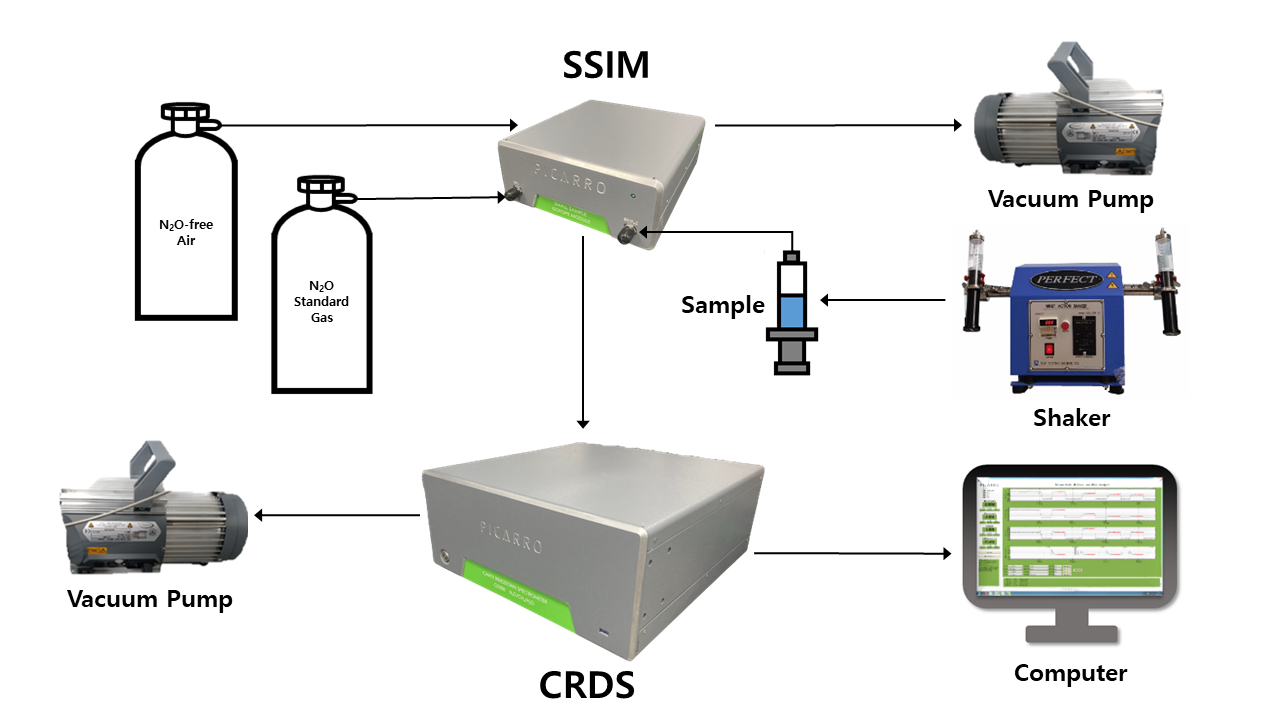


**Figure S1.** Diagram showing the cavity ring-down spectrometer (CRDS)-based N_2_O measurement system. SSIM denotes small isotope inlet module. Note that this figure was generated using Adobe Illustrator CC program (ver. 2018 and [www.adobe.com](http://www.adobe.com)) by the authors.

**
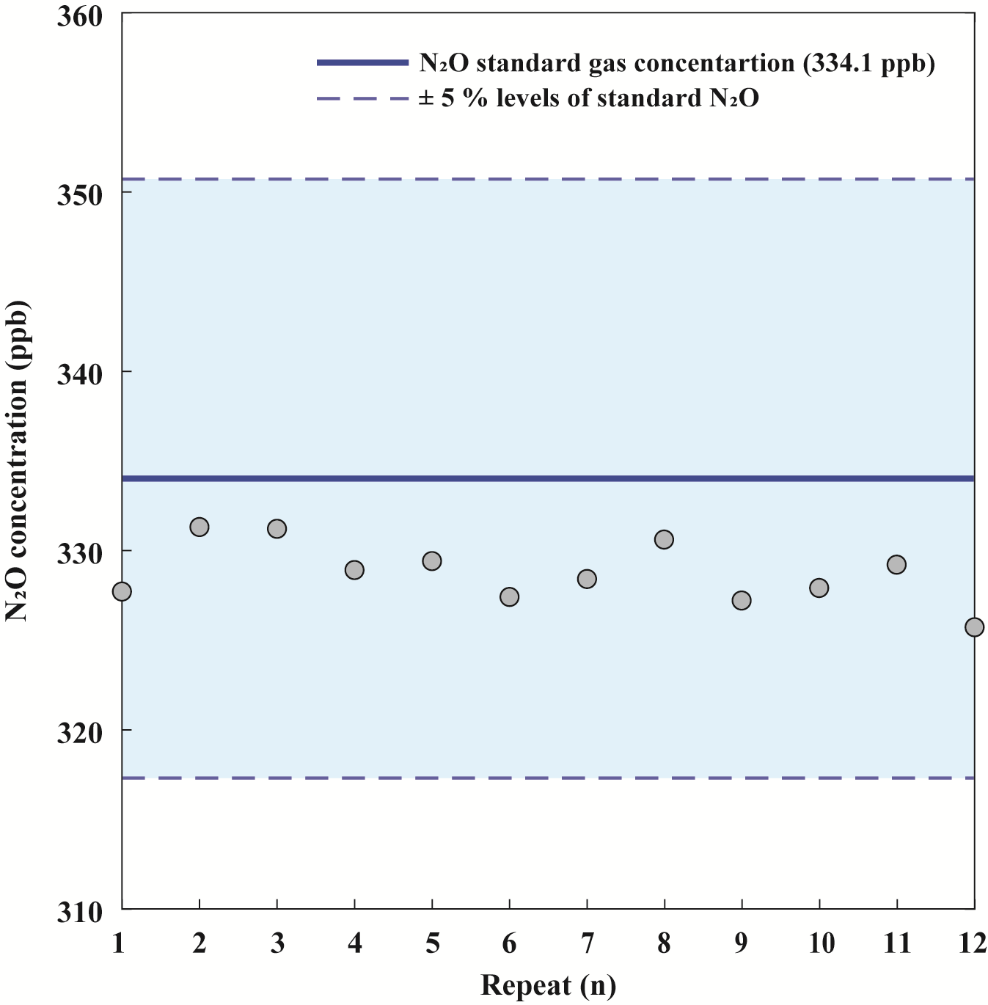
**

**Figure S2.** Repeat N_2_O measurements from CRDS system (gray circles) using a N_2_O standard gas that is officially certified as 334.1 ppb (blue solid line) by Korea Research Institute of Standards and Science (KRISS). Dotted lines represent ±5 % levels of standard N_2_O 334.1 ppb. Note that this figure was generated using MATLAB program (ver. R2019b and [www.mathworks.com](http://www.mathworks.com)).


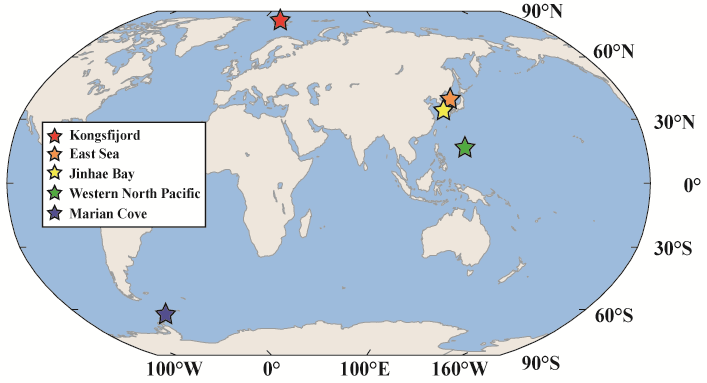


**Figure S3.** Map showing locations of duplicate samples collected at different times and locations in various marine environments (refer to Supplementary Table S3 and S4). Note that this figure was generated using MATLAB program (ver. R2019b and [www.mathworks.com](http://www.mathworks.com)).

**
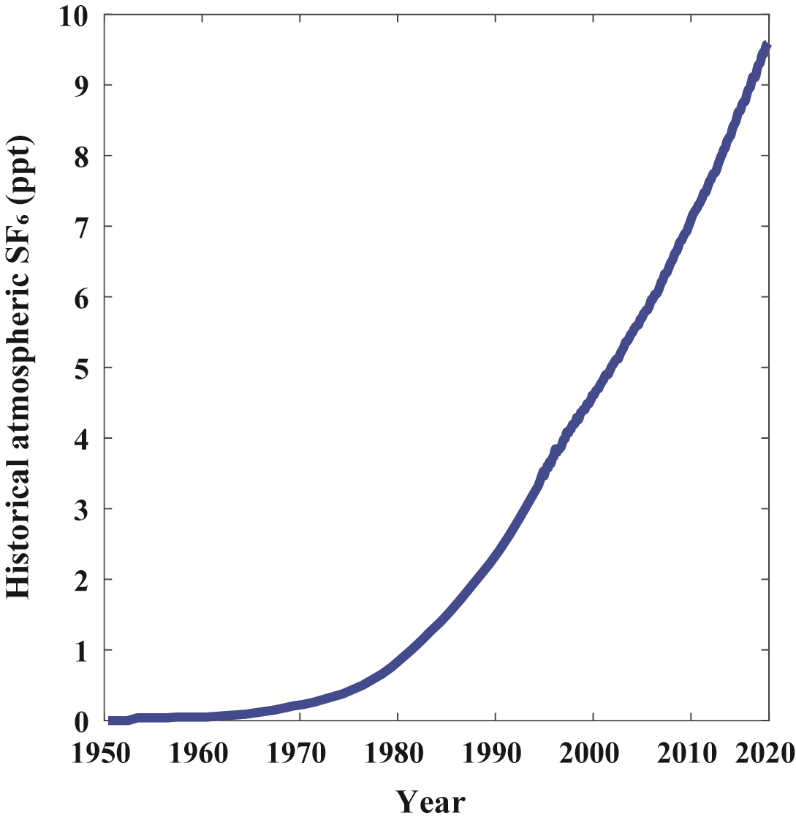
**

**Figure S4.** History of atmospheric SF_6_ levels in the northern hemisphere (source: [www.esrl.noaa.gov/](http://www.esrl.noaa.gov/)). Note that this figure was generated using MATLAB program (ver. R2019b and [www.mathworks.com](http://www.mathworks.com)).

**
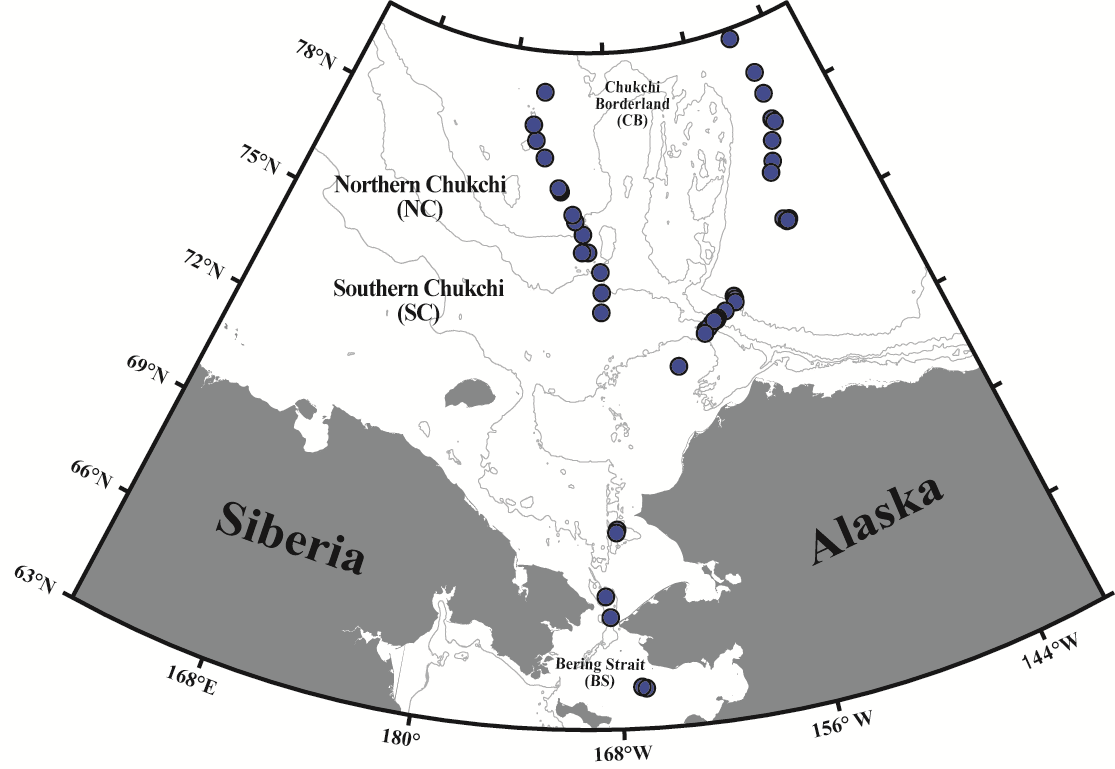
Figure S5.** A station map for the summer 2015 CLIVAR ARC01 cruise (source: [cchdo.ucsd.edu/cruise/33HQ20150809](https://cchdo.ucsd.edu/cruise/33HQ20150809)). Note that this figure was generated using MATLAB program (ver. R2019b and [www.mathworks.com](http://www.mathworks.com)).


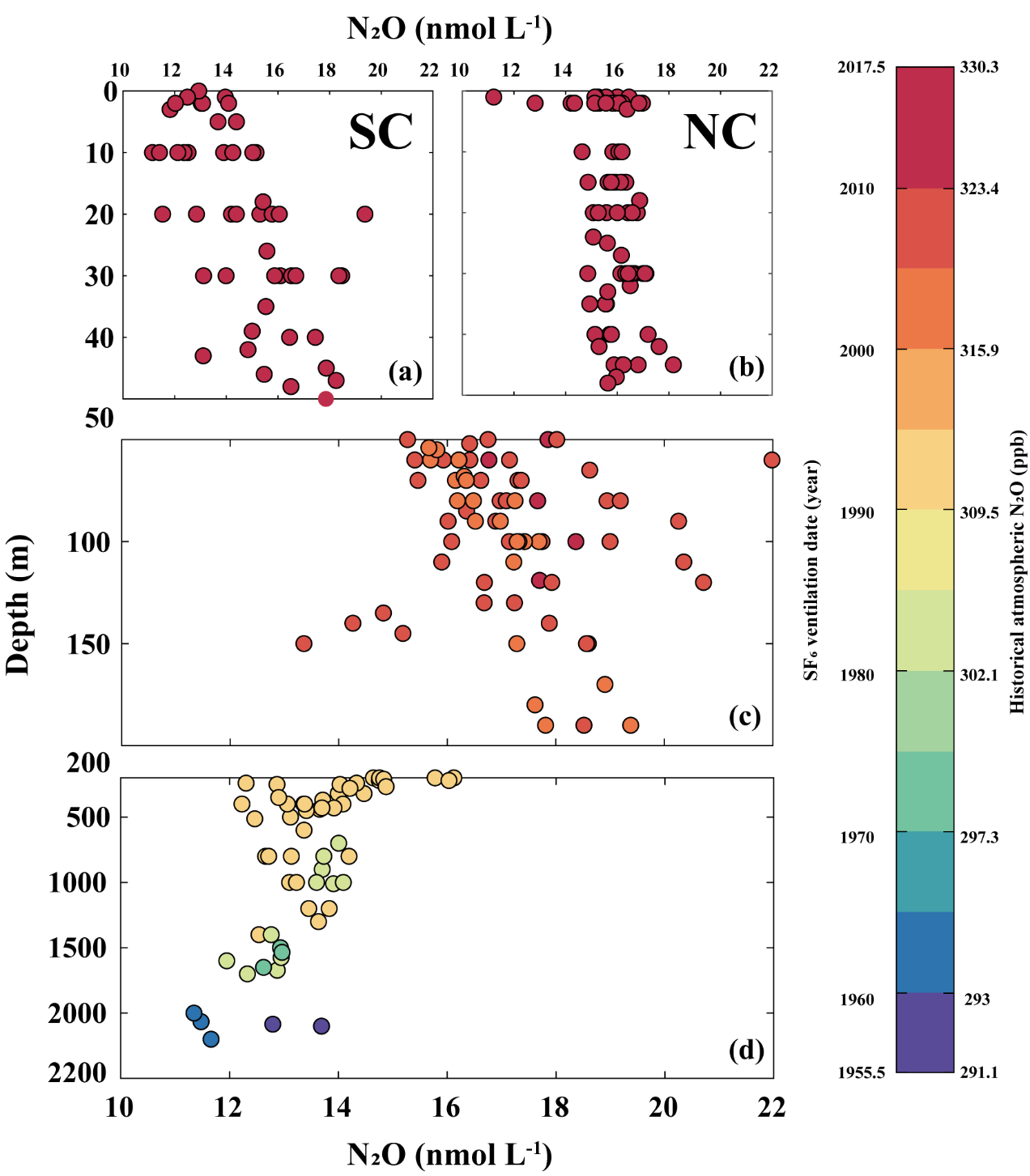


**Figure S6.** Vertical distribution of N_2_O_air_ histories in the near surface SC (**a**) and NC (**b**), intermediate waters (**c**) and deep waters (**d**) and their corresponding to the SF_6_ derived calendar years shown as color shading. Note that this figure was generated using MATLAB program (ver. R2019b and [www.mathworks.com](http://www.mathworks.com)).

**
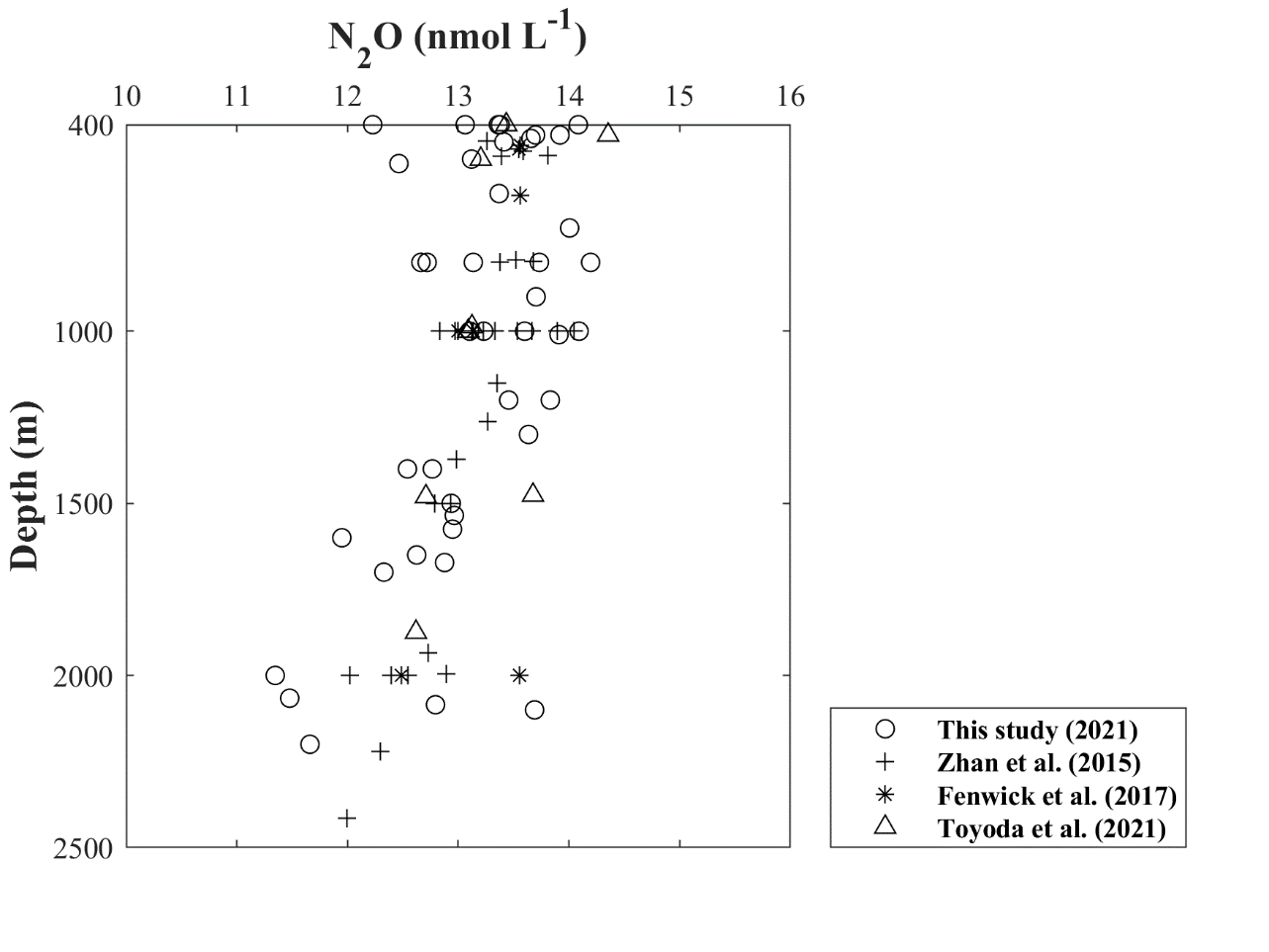
**

**Figure S7.** Vertical N_2_O distribution of this study and previous studies in the deep water of Canada Basin. Note that this figure was generated using MATLAB program (ver. R2019b and [www.mathworks.com](http://www.mathworks.com)).

**Table S1.** Observed wind speeds (m s^‒1^) at the height of 30 m (U_30_) by a windmill anemometer (05106, RM Young, USA) on the R/V Araon during the summer of 2017 WAO cruise. Wind speeds at the height of 10 m (U_10_) were estimated using the Equation (S4) (refer to Supplementary Text S1).

| **Station** | **Year** | **Date** | **Month** | **Time** | **Observed U_30_** | **Estimated U_10_** |
| --- | --- | --- | --- | --- | --- | --- |
| 1 | 2017 | 8 | 7 | 04:00–04:30 | 9.98 | 9.11 |
| 2 | 2017 | 8 | 7 | 14:15–14:45 | 8.12 | 7.41 |
| 3 | 2017 | 8 | 7 | 21:35–22:05 | 2.25 | 2.05 |
| 4 | 2017 | 8 | 8 | 00:49–01:19 | 2.21 | 2.02 |
| 5 | 2017 | 8 | 8 | 02:15–02:45 | 1.64 | 1.50 |
| 6 | 2017 | 8 | 8 | 15:06–15:36 | 3.02 | 2.76 |
| 7 | 2017 | 8 | 9 | 00:00–30:00 | 15.38 | 14.04 |
| 8 | 2017 | 8 | 9 | 08:50–09:20 | 10.91 | 9.96 |
| 9 | 2017 | 8 | 9 | 15:00–15:30 | 9.72 | 8.87 |
| 10 | 2017 | 8 | 10 | 03:07–03:37 | 13.25 | 12.09 |
| 11 | 2017 | 8 | 10 | 13:45–14:15 | 7.49 | 6.84 |
| 12 | 2017 | 8 | 10 | 23:24–23:54 | 8.80 | 8.03 |
| 13 | 2017 | 8 | 11 | 13:08–13:38 | 10.62 | 9.69 |
| 14 | 2017 | 8 | 11 | 22:28–22:58 | 11.22 | 10.24 |
| 15 | 2017 | 8 | 12 | 09:00–09:30 | 4.24 | 3.87 |
| 16 | 2017 | 8 | 12 | 19:17–19:47 | 10.05 | 9.17 |
| 17 | 2017 | 8 | 15 | 09:38–10:08 | 1.88 | 1.72 |
| 18 | 2017 | 8 | 19 | 02:23–02:53 | 1.88 | 1.72 |
| 19 | 2017 | 8 | 19 | 13:04–13:34 | 7.63 | 6.97 |
| 20 | 2017 | 8 | 20 | 01:00–01:30 | 6.74 | 6.15 |
| 21 | 2017 | 8 | 20 | 13:05–13:35 | 5.52 | 5.04 |
| 22 | 2017 | 8 | 21 | 07:40–08:10 | 2.25 | 2.05 |
| 23 | 2017 | 8 | 21 | 16:18–16:48 | 0.84 | 0.77 |
| 24 | 2017 | 8 | 22 | 01:00–01:30 | 1.56 | 1.42 |
| 25 | 2017 | 8 | 22 | 08:38–09:08 | 2.45 | 2.24 |
| 26 | 2017 | 8 | 22 | 17:28–17:58 | 3.10 | 2.83 |
| 27 | 2017 | 8 | 23 | 05:10–05:40 | 3.78 | 3.45 |
| 28 | 2017 | 8 | 23 | 14:48–15:18 | 4.76 | 4.35 |
| 29 | 2017 | 8 | 24 | 02:40–04:10 | 7.36 | 6.72 |
| 30 | 2017 | 8 | 24 | 12:00–12:30 | 8.54 | 7.80 |

**Table S2.** CRDS-based N_2_O measurements of the reference water (RW) obtained by equilibrating ambient air (N_2_O_air_ = 337.3 ppb) with seawater (T: 20.5 °C and S: 33.93 psu) for 24 hr in the lab. Equilibrated N_2_O concentration (N_2_O*_eq_* = 7.74 nmol L^‒1^) is estimated from T and S[^16^](#_ENREF_16).

| **Repeat (n)** | **Measured N_2_O_RW_**  **(nmol L^‒1^)** | **Estimated N_2_O*_eq_***  **(nmol L^‒1^)** | **Diff**  **\|N_2_O*_eq_* –N_2_O_RW_\|** | **Error**  **(%)** |
| --- | --- | --- | --- | --- |
| 1 | 7.45 | 7.74 | 0.29 | 3.75 |
| 2 | 7.98 | 7.74 | 0.24 | 3.10 |
| 3 | 8.03 | 7.74 | 0.29 | 3.75 |
| 4 | 8.02 | 7.74 | 0.28 | 3.62 |
| 5 | 8.18 | 7.74 | 0.44 | 5.68 |
| 6 | 8.12 | 7.74 | 0.38 | 4.91 |
| 7 | 7.98 | 7.74 | 0.24 | 3.10 |
| **Mean** | | | 0.31 | 3.99 |

**Table S3.** Summary of CRDS-based N_2_O measurements of the duplicate samples (represented as 1^st^ and 2^nd^) collected in different time and space from various environments. Error is the ratio of 1^st^ and Diff. values. The locations are showing Supplementary Figure S3.

| **Location** | **Region** | | **Date** | **Depth (m)** | **N_2_O (nmol L^-1^)** | | | |
| --- | --- | --- | --- | --- | --- | --- | --- | --- |
|  |  |  |  |  | **1^st^** | **2^nd^** | **Diff.**  **\|2^nd^ –1^st^\|** | **Error (%)** |
| 1 | Kongsfjord  (78.98 °N, 11.65 °E) | | Jul. 2018 | 100 | 11.01 | 11.17 | 0.16 | 1.45 |
| 2 | East Sea  (37.06°N, 131.25°E) | | Feb. 2020 | 1000 | 22.38 | 22.99 | 0.61 | 2.73 |
| 3 | Jinhae Bay  (35.98 °N , 128.48 °E) | | Jul. 2019 | 21 | 7.30 | 7.38 | 0.08 | 1.10 |
| 4 | Western North Pacific  (15.00°N, 134.50°E) | | May 2020 | 2000 | 25.30 | 25.70 | 0.40 | 1.58 |
| 5 | Marian Cove  (62.21 °N, 121.23 °W) | | Jan. 2018 | 14 | 14.76 | 14.65 | 0.11 | 0.75 |
|  | | **Mean** | | | | | 0.25 | 1.38 |

**Table S4.** Summary of CRDS-based N_2_O measurements of the duplicate samples (represented as 1^st^ and 2^nd^) from surface to bottom in East Sea and Western North Pacific. Error is the ratio of 1^st^ and Diff. values. The locations are showing Supplementary Figure S3.

| **Location** | **Region** | | **Date** | **Depth (m)** | **N_2_O (nmol L^-1^)** | | | |
| --- | --- | --- | --- | --- | --- | --- | --- | --- |
|  |  |  |  |  | **1^st^** | **2^nd^** | **Diff.**  **\|2^nd^ –1^st^\|** | **Error (%)** |
| 1 | East Sea  (37.06°N, 131.25°E) | | Aug. 2020 | 0 | 7.49 | 7.25 | 0.24 | 3.21 |
|  |  |  |  | 50 | 12.55 | 12.49 | 0.06 | 0.48 |
|  |  |  |  | 100 | 12.32 | 12.24 | 0.08 | 0.65 |
|  |  |  |  | 150 | 15.47 | 15.69 | 0.22 | 1.42 |
|  |  |  |  | 300 | 18.47 | 18.31 | 0.16 | 0.87 |
|  |  |  |  | 500 | 21.66 | 21.11 | 0.55 | 2.54 |
|  |  |  |  | 750 | 21.29 | 21.91 | 0.62 | 2.91 |
|  |  |  |  | 1000 | 19.73 | 19.77 | 0.04 | 0.20 |
|  |  |  |  | 1500 | 17.84 | 17.96 | 0.12 | 0.67 |
|  |  |  |  | 2000 | 17.18 | 16.99 | 0.19 | 1.11 |
|  |  |  |  | 2170 | 16.95 | 17.28 | 0.33 | 1.95 |
| 2 | Western North Pacific  (17.00°N, 134.50°E) | | May 2020 | 0 | 6.86 | 6.57 | 0.29 | 4.23 |
|  |  |  |  | 50 | 6.85 | 7.04 | 0.19 | 2.78 |
|  |  |  |  | 100 | 7.04 | 6.98 | 0.06 | 0.85 |
|  |  |  |  | 150 | 8.34 | 8.22 | 0.12 | 1.44 |
|  |  |  |  | 300 | 12.51 | 12.78 | 0.27 | 2.16 |
|  |  |  |  | 500 | 31.36 | 30.82 | 0.54 | 1.72 |
|  |  |  |  | 1000 | 29.83 | 29.45 | 0.38 | 1.27 |
|  |  |  |  | 2000 | 25.54 | 24.85 | 0.69 | 2.70 |
|  |  |  |  | 3000 | 22.25 | 21.63 | 0.62 | 2.79 |
|  |  |  |  | 4000 | 21.29 | 20.78 | 0.51 | 2.40 |
|  |  |  |  | 5000 | 20.88 | 20.45 | 0.43 | 2.06 |
|  |  |  |  | 5357 | 21.71 | 21.02 | 0.69 | 3.18 |
|  | | **Mean** | | | | | 0.32 | 1.89 |

**Table S5.** Histories of N_2_O_air_ for the period of 1955.5‒1975.5 were estimated from the ice core records[^17^](#_ENREF_17), and for the period of 1980.5‒2017.5 were from the atmospheric observation at the Barrow observatory (Barrow, Alaska, USA, 71.3°N, 156.6°W) (available at [www.esrl.noaa.gov](http://www.esrl.noaa.gov/)).

| **Year** | **N_2_O (ppb)** | **Data resource** |
| --- | --- | --- |
| 1955.5 | 291.1 | Firn and ice core |
| 1960.5 | 293.0 | Firn and ice core |
| 1965.5 | 295.0 | Firn and ice core |
| 1970.5 | 297.3 | Firn and ice core |
| 1975.5 | 299.7 | Firn and ice core |
| 1980.5 | 301.6 | Field observation |
| 1985.5 | 304.7 | Field observation |
| 1990.5 | 309.1 | Field observation |
| 1995.5 | 311.7 | Field observation |
| 2000.5 | 315.9 | Field observation |
| 2005.5 | 319.4 | Field observation |
| 2010.5 | 323.4 | Field observation |
| 2015.5 | 328.5 | Field observation |
| 2017.5 | 330.3 | Field observation |

**Table S6.** Major water masses identified in the study area during the summer of 2017.

| **Water mass** | **Acronym** | **Depth** | **Physicochemical**  **characteristics** | **References** |
| --- | --- | --- | --- | --- |
| Fresh Water | FW | <~50 m | <~30 psu | Coachman & Aagaard, (1974)[^18^](#_ENREF_18);  Aagaard et al. (1981)[^19^](#_ENREF_19);  Steele et al. (2004)[^20^](#_ENREF_20);  Woodgate et al. (2005b)[^21^](#_ENREF_21);  Talley et al. (2011)[^22^](#_ENREF_22);  Itoh et al. (2015)[^23^](#_ENREF_23);  Danielson et al. (2017)[^24^](#_ENREF_24);  Corlett & Pickart, (2017)[^25^](#_ENREF_25);  This study (2021) |
| Pacific Summer Water | PSW | <~50 m | >0 °C (max PT)  ~30‒33.6 psu |  |
| Pacific Winter Water | PWW | ~50–200 m | <0 °C  ~31.5‒33.6 psu  (min PT, DO, N^*^, and  max N&P) |  |
| Atlantic Water | AW | ~200–1000 m | ~0‒2 °C  >~34.9 psu  (max PT and N^*^) |  |
| Arctic Bottom Water | ABW | below ~1000 m | <0 °C  >~34.95 psu  (max N^*^) |  |

**Table S7.** Deep N_2_O concentrations and measuring methods of this study and previous studies.

| **References** | **Sampling period** | **N_2_O in the deep water of  Canada Basin** | **Method for extracting dissolved N_2_O** | **Analysis equipment** |
| --- | --- | --- | --- | --- |
| This study (2021) | August 2017 | 13.4 ± 0.5^a^ (400 to 1000 m) 12.2 ± 1.0^a^ (below 2000 m) | Headspace | CRDS |
| Zhan et al. (2015)[^26^](#_ENREF_26) | July to September 2010 | 13.5^a^ (400 to 1000 m) 12.5^a^ (below 2000 m) | Headspace | Gas Chromatograph |
| Fenwick et al. (2017)[^27^](#_ENREF_27) | July to October 2015 | 13 ± 0.5^a^ (below 2000 m) | Purge and Trap | Gas Chromatograph |
| Toyoda et al. (2021)[^28^](#_ENREF_28) | September 2014 &  September to October 2015 | 12^b^ (below 1000 m) | Purge and Trap | Gas Chromatograph |
| ^a^Unit is nmol L^-1^. ^b^Unit is nmol kg^-1^. | | | | |

**Table S8.** Estimated N_2_O fluxes during the summer of 2017 in the western Arctic Ocean. Three *k_w_* models of Wanninkhof and McGillis (1999; WM_1999_)[^2^](#_ENREF_2), Nightingale et al. (2000; N_2000_)[^3^](#_ENREF_3), and Wanninkhof (2014; W_2014_)[^4^](#_ENREF_4) were applied to estimate N_2_O fluxes in the Equation (3), and then used mean values averaged from the three models (refer to Supplementary Text S1).

| **Region** | **Station** | **N_2_O Flux (μmol N_2_O m**^‒^**^2^ d**^‒^**^1^)** | | | | |
| --- | --- | --- | --- | --- | --- | --- |
|  |  | **W&M_1999_** | **N_2000_** | **W_2014_** | **Mean** | **STD** |
| SC | 1 | 8.81 | 8.43 | 8.58 | 8.61 | 0.19 |
|  | 2 | 2.48 | 3.01 | 2.97 | 2.82 | 0.30 |
|  | 3 | 0.07 | 0.42 | 0.29 | 0.26 | 0.18 |
|  | 4 | 0.08 | 0.52 | 0.36 | 0.32 | 0.22 |
|  | 5 | 0.02 | 0.21 | 0.12 | 0.12 | 0.10 |
|  | 6 | 0.10 | 0.40 | 0.31 | 0.27 | 0.15 |
|  | 7 | 5.40 | 3.18 | 3.41 | 4.00 | 1.22 |
|  | 8 | 2.41 | 2.08 | 2.15 | 2.21 | 0.17 |
|  | 9 | 1.85 | 1.82 | 1.85 | 1.84 | 0.02 |
| Mean | | | | | 2.27 | 0.28 |
| NC | 10 | -2.45 | -1.70 | -1.80 | -1.98 | 0.41 |
|  | 11 | -1.35 | -1.80 | -1.75 | -1.63 | 0.25 |
|  | 12 | -3.19 | -3.52 | -3.52 | -3.41 | 0.19 |
|  | 13 | -4.61 | -4.11 | -4.22 | -4.31 | 0.26 |
|  | 14 | -4.63 | -3.88 | -4.01 | -4.17 | 0.40 |
|  | 15 | -0.41 | -1.11 | -0.95 | -0.82 | 0.37 |
|  | 16 | -3.81 | -3.61 | -3.68 | -3.70 | 0.10 |
|  | 17 | -0.04 | -0.29 | -0.19 | -0.17 | 0.13 |
|  | 18 | -0.03 | -0.27 | -0.17 | -0.16 | 0.12 |
|  | 19 | -1.78 | -2.33 | -2.27 | -2.13 | 0.30 |
|  | 20 | -0.42 | -0.63 | -0.60 | -0.55 | 0.11 |
|  | 21 | -0.03 | -0.06 | -0.05 | -0.05 | 0.02 |
|  | 22 | -0.02 | -0.16 | -0.11 | -0.10 | 0.07 |
|  | 23 | -0.01 | -0.19 | -0.08 | -0.09 | 0.09 |
|  | 24 | -0.002 | -0.02 | -0.01 | -0.01 | 0.01 |
|  | 25 | -0.11 | -0.61 | -0.43 | -0.38 | 0.25 |
|  | 26 | -0.04 | -0.15 | -0.12 | -0.10 | 0.06 |
|  | 27 | -0.003 | -0.01 | -0.01 | -0.01 | 0.004 |
|  | 28 | -0.50 | -1.17 | -1.03 | -0.90 | 0.35 |
|  | 29 | -0.70 | -0.95 | -0.92 | -0.86 | 0.14 |
|  | 30 | -1.26 | -1.44 | -1.43 | -1.38 | 0.10 |
| Mean | | | | | -1.28 | 0.18 |

**Supplementary References**

1 Wanninkhof, R. Relationship between wind speed and gas exchange over the ocean. *Journal of Geophysical Research: Oceans* **97**, 7373-7382 (1992).

2 Wanninkhof, R. & McGillis, W. R. A cubic relationship between air‐sea CO_2_ exchange and wind speed. *Geophysical Research Letters* **26**, 1889-1892 (1999).

3 Nightingale, P. D. *et al.* In situ evaluation of air‐sea gas exchange parameterizations using novel conservative and volatile tracers. *Global Biogeochemical Cycles* **14**, 373-387 (2000).

4 Wanninkhof, R. Relationship between wind speed and gas exchange over the ocean revisited. *Limnology and Oceanography: Methods* **12**, 351-362 (2014).

5 PARK, M.-K. & PARK, S. Recent Technological Advances in Optical Instruments and Future Applications for in Situ Stable Isotope Analysis of CH_4_ in the Surface Ocean and Marine Atmosphere. *The Sea* **23**, 32-48 (2018).

6 Siedler, G. & Peters, H.  *Physical properties (general) of sea water.* (Springer, 1986).

7 Holmes, J. D. *Wind loading of structures*. (CRC press, 2018).

8 Troen, I. & Lundtang Petersen, E. European wind atlas. (1989).

9 Rapson, T. D. & Dacres, H. Analytical techniques for measuring nitrous oxide. *TrAC Trends in Analytical Chemistry* **54**, 65-74 (2014).

10 Wilson, S. T. *et al.* An intercomparison of oceanic methane and nitrous oxide measurements. *Biogeosciences* **15**, 5891-5907 (2018).

11 Crosson, E. A cavity ring-down analyzer for measuring atmospheric levels of methane, carbon dioxide, and water vapor. *Applied Physics B* **92**, 403-408 (2008).

12 Erler, D. V. *et al.* Applying cavity ring‐down spectroscopy for the measurement of dissolved nitrous oxide concentrations and bulk nitrogen isotopic composition in aquatic systems: Correcting for interferences and field application. *Limnology and Oceanography: Methods* **13**, 391-401 (2015).

13 Troncoso, M., Garcia, G., Verdugo, J. & Farías, L. Toward high-resolution vertical measurements of dissolved greenhouse gases (nitrous oxide and methane) and nutrients in the eastern South Pacific. *Frontiers in Marine Science* **5**, 148 (2018).

14 Zhan, L. *et al.* A fully automatic system for underway N_2_O measurements based on cavity ring-down spectroscopy. *International Journal of Environmental Analytical Chemistry* **98**, 709-724 (2018).

15 Roberts, H. M. & Shiller, A. M. Determination of dissolved methane in natural waters using headspace analysis with cavity ring-down spectroscopy. *Analytica chimica acta* **856**, 68-73 (2015).

16 Weiss, R. & Price, B. Nitrous oxide solubility in water and seawater. *Marine chemistry* **8**, 347-359 (1980).

17 Macfarling Meure, C. *et al.* Law Dome CO_2_, CH_4_ and N_2_O ice core records extended to 2000 years BP. *Geophysical Research Letters* **33** (2006).

18 Coachman, L. K. & Aagaard, K. in *Marine geology and oceanography of the Arctic seas* 1-72 (Springer, 1974).

19 Aagaard, K., Coachman, L. & Carmack, E. On the halocline of the Arctic Ocean. *Deep Sea Research Part A. Oceanographic Research Papers* **28**, 529-545 (1981).

20 Steele, M. *et al.* Circulation of summer Pacific halocline water in the Arctic Ocean. *Journal of Geophysical Research: Oceans* **109** (2004).

21 Woodgate, R. A., Aagaard, K. & Weingartner, T. J. A year in the physical oceanography of the Chukchi Sea: Moored measurements from autumn 1990–1991. *Deep Sea Research Part II: Topical Studies in Oceanography* **52**, 3116-3149 (2005).

22 Talley, L. D. *Descriptive physical oceanography: an introduction*. (Academic press, 2011).

23 Itoh, M. *et al.* Water properties, heat and volume fluxes of Pacific water in Barrow Canyon during summer 2010. *Deep Sea Research Part I: Oceanographic Research Papers* **102**, 43-54 (2015).

24 Danielson, S. L. *et al.* A comparison between late summer 2012 and 2013 water masses, macronutrients, and phytoplankton standing crops in the northern Bering and Chukchi Seas. *Deep Sea Research Part II: Topical Studies in Oceanography* **135**, 7-26 (2017).

25 Corlett, W. B. & Pickart, R. S. The Chukchi slope current. *Progress in Oceanography* **153**, 50-65 (2017).

26 Zhan, L., Chen, L., Zhang, J. & Li, Y. A vertical gradient of nitrous oxide below the subsurface of the Canada Basin and its formation mechanisms. *Journal of Geophysical Research: Oceans* **120**, 2401-2411 (2015).

27 Fenwick, L. *et al.* Methane and nitrous oxide distributions across the North American Arctic Ocean during summer, 2015. *Journal of Geophysical Research: Oceans* **122**, 390-412 (2017).

28 Toyoda, S. *et al.* Distribution and Production Mechanisms of N_2_O in the Western Arctic Ocean. *Global Biogeochemical Cycles* **35**, e2020GB006881, doi:https://doi.org/10.1029/2020GB006881 (2021).
